# Propolis and Its Gastroprotective Effects on NSAID-Induced Gastric Ulcer Disease: A Systematic Review

**DOI:** 10.3390/nu13093169

**Published:** 2021-09-11

**Authors:** Porfirio Alonso Ruiz-Hurtado, Leticia Garduño-Siciliano, Pilar Domínguez-Verano, Daniela Balderas-Cordero, Gustavo Gorgua-Jiménez, Octavio Canales-Álvarez, María Margarita Canales-Martínez, Marco Aurelio Rodríguez-Monroy

**Affiliations:** 1 Laboratorio de Toxicología de Productos Naturales, Departamento de Farmacia, Instituto Politécnico Nacional, Escuela Nacional de Ciencias Biológicas, Av. Wilfrido Massieu, Gustavo A. Madero, Ciudad de México 07738, Mexico; alonsoruiz55@gmail.com (P.A.R.-H.); lsicilia@hotmail.com (L.G.-S.); 2 Laboratorio de Investigación Biomédica en Productos Naturales, Carrera de Medicina, UNAM, FES-Iztacala, Avenida de los Barrios Número 1, Colonia Los Reyes Iztacala, Estado de México 54090, Mexico; pilardomver@hotmail.com (P.D.-V.); daniebalcord@hotmail.com (D.B.-C.); ggorgua@ciencias.unam.mx (G.G.-J.); octaviocanalesa@gmail.com (O.C.-Á.); 3 Laboratorio de Genética, Departamento de Farmacia, Instituto Politécnico Nacional, Escuela Nacional de Ciencias Biológicas, Av. Wilfrido Massieu, Gustavo A. Madero, Ciudad de México 07738, Mexico; 4 Laboratorio de Farmacognosia, UBIPRO, UNAM, FES-Iztacala, Avenida de los Barrios Número 1, Colonia Los Reyes Iztacala, Estado de México 54090, Mexico; dra.margaritacanales@gmail.com

**Keywords:** NSAIDs, propolis, gastric ulcer disease, phenolic compounds

## Abstract

Gastric ulcer disease induced by the consumption of NSAIDs is a major public health problem. The therapy used for its treatment causes adverse effects in the patient. Propolis is a natural product that has been used for the treatments of different diseases around the world. Nevertheless, there is little information about the activity of propolis in gastric ulcers caused by treatment with NSAIDs. Therefore, this review evaluates and compares the gastroprotective potential of propolis and its function against NSAID-induced gastric ulcers, for which a systematic search was carried out in the PubMed and ScienceDirect databases. The main criteria were articles that report the gastroprotective activity of propolis against the damage produced by NSAIDs in the gastric mucosa. Gastroprotection was related to the antioxidant, antisecretory, and cytoprotective effects, as well as the phenolic compounds present in the chemical composition of propolis. However, most of the studies used different doses of NSAIDs and propolis and evaluated different parameters. Propolis has proven to be a good alternative for the treatment of gastric ulcer disease. However, future studies should be carried out to identify the compounds responsible for these effects and to determine their potential use in people.

## 1. Introduction

The most commonly prescribed drugs for pain and inflammation treatment are non-steroidal anti-inflammatory drugs (NSAIDs). Their beneficial properties have been used in the prevention and treatment of diverse disorders because of the general perception of their safety [1,2,3]. An example of this is the long-term use of aspirin, which decreases the risk of cardiovascular diseases, as well as ischemic stroke, colorectal cancer, and myocardial infarction [3]. Nevertheless, NSAIDs have limitations because of side effects that principally affect the gastrointestinal (GI) tract. The increased risk of developing hemorrhage, stroke, and GI bleeding, and the formation of gastric lesions are well known, and therefore, gastric ulcer formation is related to the response to stress; furthermore, treatment with NSAIDs decreases gastric ulcer healing [2,3].

### 1.1. NSAIDs Adverse Effects

Therapy with NSAIDs causes complications in the GI tract in about 40% of the population and in 20% to 30% in chronic users; a relevant fact is that 13% of patients with gastric ulcers are associated with the use of NSAIDs. It is known that the patients with chronic administration of these drugs increase the risk of developing gastroduodenal mucosal erosion (35–60%), ulceration (10–25%), and severe hemorrhages and perforation (<1%). Additionally, epidemiological studies have shown that NSAIDs enhance the risk of complications such as bleeding and perforation; some patients even require hospitalization, and 30% of cases die. These events have a negative impact on the quality of life of patients; moreover, gastric ulcers are a risk factor for the development of gastric cancer [3,4].

Nevertheless, GI damage is not only caused by long-term or chronic exposure to NSAIDs, acute treatments can produce damage in GI tissue, such as aspirin, which causes damage in the first 60 min, and is evidenced in an endoscopic examination where extensive intramucosal petechial hemorrhage and erosion are evidenced. A hypothesis proposes that NSAIDs induce inhibition of platelet aggregation, and, therefore, the topical and systemic mucosal damage in the stomach is amplified. Moreover, different factors can increase the occurrence of GI disorders, such as age, osteoarthritis, duration of NSAIDs treatment, previous ulcer disease, and current cotreatment with corticosteroids; another factor that could increase the risk of NSAID-induced ulcers is female sex [1]. A higher risk factor that aggravates the potential of NSAIDs-induced GI damage is infection with *Helicobacter pylori* [2,3,4]. When this bacterium infects the gastric mucosa, the harmful effects associated with it in the mucosa, such as hemorrhagic erosion, neutrophil infiltration, lymphoid follicles, and epithelium damage are aggravated by indomethacin (an NSAID), but it does not have an effect on the increase in bacterial numbers [3].

### 1.2. Mechanism of NSAIDs to Induce GI Damage

GI damage by NSAIDs is induced by biochemical alterations in the epithelial cells of the gastric mucosa, although is important to take into account that the cellular damage caused by the different NSAIDs varies according to the potency of their GI side effects [3,5]. As NSAIDs (weak acids) are lipid-soluble, they are capable of penetrating epithelial cells of the gastric mucosa and then inhibit two isoforms of cyclooxygenases (COX) COX-1 and COX-2; both enzymes are key in the prostaglandin synthesis from arachidonic acid that is derived from the cellular membrane phosphorylation [3,6]. Interestingly, the inhibition of COX enzymes is the basis of the anti-inflammatory action of NSAIDs, but, contradictory to this, the same inhibitory effect is responsible for their adverse effects in the GI tract as well as inhibition of the platelet aggregation [1].

Molecules synthesized in COX isoforms (prostaglandins (PGE_2_), thromboxane A2, leukotrienes, and prostacyclin (PGI_2_)) have a similar structure and their own identical biological actions; nevertheless, both COX-1 and COX-2 generate a different pattern of prostaglandins and thromboxane; moreover, both enzymes have different tissue localization, distribution, and regulation; therefore, the activation of COX enzymes results in different biological responses, which is crucial for the beneficial or adverse effects of COX inhibitors such as NSAIDs [1,3,5]. The classical hypothesis according to COX isoforms indicates that COX-1 is expressed constitutively in most tissues including the stomach, and COX-2 appears to be expressed due to the response to damage in different tissues; therefore, it is referred to as an inducible isoform [1,2,3,4].

The above is supported because COX-1 has a housekeeping function because the normal gastric tissue produces the prostaglandins that are related to platelet function, hemostasis regulation, the regulation of acid secretion, and gastric mucosal protection. By contrast, prostaglandins produced by COX-2 induce inflammation, pain, and fever, but at the same, prostaglandins are related to cell proliferation, angiogenesis promotion, and mucosal integrity restoration [5,6]. However, COX-2 is known to have a complex biological role and is not only involved in mediating inflammation and pain, this enzyme is not only inducible in inflammatory responses but is also constitutive of normal non-inflamed tissues, such as the GI mucosa, since there are detectable amounts of COX-2 mRNA and proteins. Additionally, immunohistochemical studies expose COX-2 localization in diverse cell types, such as endothelial cells, surface epithelial cells, mesenchymal cells, and parietal cells, and in vitro studies have localized this enzyme in epithelial cells from healthy rats’ gastric mucosa [1].

Although it has been reported that NSAID-induced gastric ulcers only occur as a result of COX-1 inhibition, there is evidence that GI damage is produced by inhibition of both COX-1 and COX-2; the reason for this is that when performing a selective inhibition of COX-1 or COX-2 there is no gastric damage. This demonstrates that GI damage caused by NSAIDs is related to both COX enzymes, and not only to COX-1 inhibition. Moreover, inhibition of COX-1 overexpresses COX-2 and the prostaglandins that produce help in the maintenance and gastroprotection of the gastric mucosa in the absence or reduction of prostaglandins produced by COX-1 [2,4,5].

The inhibition of COX-1 and COX-2 reduces the mucosal blood flow, mucus, and bicarbonate secretion, causes vascular injury and leucocyte accumulation, and decreases cell turnover; hence, microvascular damage plays a role central in NSAID-induced mucosal damage. Prostaglandin inhibition increases neutrophil adherence in the vascular endothelium in the gastric tissue [1,6]. Conversely, PGE_2_ and PGI_2_ are key molecules in the gastroprotective process because they reduce acid secretion, increase the thickness of the mucus layer, and improve the blood flow of the mucosa [5]. Moreover, nitric oxide (NO) has the capacity to counter the harmful effects of prostaglandin inhibition by NSAIDs such as the reduction in the blood flow and the increase in the neutrophil adhesion in the microvascular endothelium of the gastric mucosa [1,6].

### 1.3. Gastric Ulcer Disease

The changes produced by NSAIDs in the gastric mucosa epithelium permeability produce lesions, caused by gastric mucus reduction, and, then, the diffusion and damages caused by gastric acid in the stomach; these lesions are knowing as gastric ulcers, which are defined as an injury in the gastric mucosa [6,7,8,9,10]. Gastric ulcer disease is traditionally also named peptic ulcer disease (PDU), but the latter refers to ulcers that can be in the stomach and the proximal duodenum (which are the most common organs affected by the secretion of pepsin and gastric acid), although the lower esophagus, distal duodenum, and jejunum can be affected but to a lesser extent [8]. 

The diagnosis of PDU is based on the occurrence of different symptoms, endoscopies, or barium contrast and tests for *H. pylori*; nevertheless, the symptoms are nonspecific, although they can present as a combination of conditions that allow it to be diagnosed [2,11,12]. Only in the United States of America, PDU affects 500,000 people, and 70% of the cases are presented in the age group that includes those between 25 and 64 years old [8]; worldwide, it is estimated to affect 50% of the population, and the gastric ulcer-specific disease affects 5–10% of the world’s population [8,9].

### 1.4. Actual Therapy for the Treatment of Gastric Ulcer Disease and Its Side Effects

Normally, the stomach is able to resist diverse noxious conditions, such as gastric secretions, alcohol, foodstuffs with varying temperatures, and osmolarities, due to its capacity to repair itself, in particular, due to its ability to repair the mucosal layer exposed to the harmful environment [8]. Nevertheless, when this capacity is reduced or inhibited by the consumption of NSAIDs, it is necessary to resort to the use of medicines for the treatment of gastric ulcer disease. Most of the strategies used to treat this pathology include the use of proton pump inhibitors (PPIs), H_2_ receptor antagonists (H_2_RAs), the use of drugs that stimulate the proliferation of the mucosal barrier or prostaglandin analogs, and the use of COX-2-selective NSAIDs, as well as antibiotics used to eliminate the *H. pylori* infection (in necessary cases) [4,10]. 

H_2_RAs were developed in the late 1970s, and these drugs cancel the acid-secreting effects of histamine because the H_2_RAs competitively binds to histamine H_2_ receptors on the basolateral plasma membrane of the parietal cells, resulting in inhibition of gastric acid secretion, mainly during the night in which histamine-stimulated acid secretion is important. On the other hand, H_2_RAs do not inhibit gastrin or the acetylcholine-induced stimulation of gastric acid secretion, especially during the post-prandial period. The suppressive effect of the acid secretion of H_2_RAs quickly appears when the first dose is administered and its plasma concentration increases; however; after two weeks, the activity of the receptor antagonist decrease due to a tolerance phenomenon as a consequence of the repeated administration [11]. Thus, the standard doses of H_2_RAs cannot reduce the risk of gastric ulcer [9].

Historically, PPIs have been available since 1989 with the discovery of omeprazole, from which different available forms of this type of drugs have been developed, such as esomeprazole, lansoprazole, pantoprazole, rabeprazole, and dexlansoprazole. Previously, with the aim of enhancing their inhibitory activity, rabeprazole, for example, forms a partially reversible bond with the proton pump and is activated at a broader range of pH in the stomach, causing two- to three-fold greater antisecretory activity than that of omeprazole, and, as it is not metabolized through an enzymatic pathway, it has little interaction with other medications [11,13]. Moreover, PPIs are the most used drugs for the management of gastric ulcers [9,13].

PPIs are drugs known for their capacity to inhibit gastric acid secretions; this property is related to the ability to bind H^+^/K^+^-ATPase of the acid secretory cells (parietal cells) in the gastric mucosa [11,13,14] because PPIs are lipophilic weak bases that cross the parietal cell membrane and enter the acid parietal cell canaliculus. The acid environment of the parietal cells protonates the PPIs and produces the activated sulphenamide form of the drug and then irreversible binds covalently to the proton pump-ATPase enzyme (H^+^/K^+^-ATPase); this has the consequence that the acid secretion of the proton pump is inhibited. Therefore, the parietal cells have to produce new proton pump-ATPase enzymes to recover their acid secretion activity [13]. PPIs are commonly administered as enteric-coated tablets or capsules that arrive in the stomach intact and are absorbed in the proximal small bowel; this form has a short plasma half-life (about two hours) but, curiously, has a longer duration of action due to the mechanism of action [11,13].

H2RAs and PPIs have similar side effects, which include headache, nausea, abdominal pain, and diarrhea, and these are related to the acid suppression of PPIs, which alters the bacterial content of the gut [13]. Moreover, PPI side effects can be divided into two groups: acid inhibition-related adverse effects and the side effects unrelated to acid inhibition [4,11]. The majority of the side effects are observed in the first group during long-term treatment; in the second group, the side effects can be present in both long-term and short-term treatments [11].

The adverse effects of PPIs unrelated to acid inhibition include allergic reaction to drug chemicals, collagenous colitis, acute intestinal nephritis, chronic kidney disease, drug interaction during the activation and/or degradation phase in the liver, dementia, and other conditions that could be associated with the long-term use of PPIs, such as the increase in risks of cerebral ischemic disease, ischemic cardiac disease unrelated to clopidogrel administration, and even decreased life expectancy. The studies that present these findings suggest the need for research that reveals the true risk of using PPIs with these diseases [11].

In contrast, the known side effects caused by the acid inhibition of PPIs include the risk of pneumonia, changes in the gut microbiome and small intestinal bacterial overgrowth, and increased GI infections caused by *Salmonella*, *Campylobacter*, and *Clostridium*; additionally, PPIs treatment increases the plasma gastrin concentration by increasing intragastric pH and can increase the risk of developing gastric neuroendocrine and carcinoid tumors; hypergastrinemia as a result of long-term use of PPIs increases the proliferation of the gastric mucosal stem system in the neck area of the gastric fundic glands resulting in a gastric fundic mucosal hypertrophy; moreover, this causes a decrease in the absorption of micronutrients (magnesium, iron, calcium, and vitamin B12), gastric fundic polyps, gastric and colon cancer, and, finally, spontaneous bacterial peritonitis and hepatic encephalopathy, without taking into account the interactions that PPIs can have with others medications [3,9,11].

### 1.5. Folk Medicine and Its Relevance as a Source of New Treatments

Over time, different cultures around the world have demonstrated a deep understanding of the environment and its ecology, preserving and transmitting various forms and effective procedures applicable to plants, mainly to solve and prevent community health problems [15,16]. The World Health Organization (WHO) defines folk medicine (or traditional medicine) as “the sum total of knowledge, skill, and practices based on the theories, beliefs, and experiences indigenous to different cultures, whether explicable or not, used in the maintenance of health as well as in the prevention, diagnosis, improvement or treatment of physical and mental illness” [17]. Within folk medicine, the use of natural products has become more popular in recent years, not only due to availability and tradition but also in the scientific field in order to find new alternatives for the treatment of different diseases [15,16,17].

The side effects of the different types of medication for the treatment of gastric ulcer disease, in addition to the fact that some populations do not have access to the standard therapy, make the use and of natural products relevant since most of these products are seen as the main reservoir of potential new drugs [4,18,19,20]. Extracts derived from natural products are the most significant sources of new drugs; some have promising bioactive components for the treatment of gastric ulcers, especially those with an antioxidant capacity [4,21,22]. Natural products have been used as medications for the treatment of diverse diseases throughout practically all of human history based on trial-and-error tests [19,20].

Usually, therapies from natural products have been considered an option for religious and low-income people, and, therefore, many people think that natural products do not contain any pharmaceutic value; however, 80% of medical drugs after the industrial revolution were isolated from plant compounds and natural products, such as morphine, which was isolated from opium in the 19th century. However, the role of natural products remains predominant because 60% of anticarcinogenic compounds and 75% of the drugs used for the treatment of infectious diseases are derived from them [23,24].

Of the 252 drugs considered basic and essential by the WHO, 11% are derived from plants, and a significant number of synthetic drugs are obtained from natural precursors. Some drugs derived from plants are digoxin from *Digitalis* spp., quinine and quinidine from *Cinchona* spp., vincristine and vinblastine from *Catharanthus roseus*, atropine from *Atropa belladonna*, morphine and codeine from *Papaver somniferum* [24]. Therefore, the WHO considers the research of natural products from folk medicine used for the treatment of different diseases to be an essential and high-priority field of study in health programs [24,25].

### 1.6. Propolis: An Old Known Substance with Gastroprotective Potential

Among the natural products, propolis stands out due to its extensive use from the year 300 A.C. to the present [26,27,28,29]. Propolis is the name for the resinous substance, also named glue, made by honeybees (*Apis mellifera*); this term derivates from Greek *pro* (“in front of” or “at the entrance of”) and *polis* (“community” or “city”); thus, its significance would be “in defense of the hive”. The agriculture department of the United States of America describes propolis as a gum collected by honeybees from different plants, and its color varies from light yellow to dark brown and can stain the artificial hives where honeybees are maids, and, moreover, propolis can be found in the honey that they produce [26,27,30].

Propolis is a strong adhesive that honeybees make with the dry plant material that they collect and enrich with salivary secretions, and, finally, when the raw material is transformed, they use it to seal the holes in their hives and to insulate and smooth the internal walls and protect the entrance from intruders. Moreover, propolis is important because it is used to protect the colony from diseases and cover corpses to prevent their decay into the honeycomb [26,27,29].

For a long time, propolis has been employed in folk medicine for its antiseptic, antifungal, antibacterial, antiviral, anti-inflammatory, and antioxidant properties. Currently, applications of propolis include preparations for sale for the treatment of different illnesses, for example, diseases of the upper respiratory tract, the common cold, and flu-like infections, among others, as well as dermatological preparations useful in wound healing, the treatment of burns, acne, herpes simplex and genital, and neurodermatitis. Additionally, propolis is used in mouthwashes and toothpaste to prevent cavities and treat gingivitis and stomatitis. Most of these preparations are based on propolis ethanolic extracts [27,28].

The chemical composition of propolis varies according to the geographic zone origin, and more than 300 compounds have been identified in it such as polyphenols (flavonoids, phenolic acids, and steres), terpenoids, steroids, and amino acids; among them, flavonoids are widely present in them, such as pinocembrin, acacetin, chrysin, rutinin, catechin, naringenin, galangenin, luteolin, kaempferol, naringin, and quercetin [29,31,32]. It has been suggested that some flavones are modified by honeybee enzymes, probably when the raw material is collected to make propolis. However, it is important to note the fact that the chemical composition of propolis depends directly on the flora at the bee collection site as well as the species of bee that produces the propolis [27,29,31]. Due to the above, the biological properties of propolis are usually different; despite this, most propolis shares a great similarity in its overall nature as secondary metabolites; crude propolis is composed of 50% resins (considered as the phenolic fraction), 30% wax, 10% essential oils, 5% pollen, and 5% of various inorganic compounds such magnesium, nickel, calcium, iron, and zinc [26,27,31,33,34,35].

The literature has reported the antimicrobial activities of propolis ethanolic extracts of different countries such as the United States of America, Brazil, Taiwan, France, Turkey, Chile, and Mexico [36,37,38,39,40,41,42]. It highlights that Brazilian propolis collected in different seasons does not present significant differences in terms of its antibacterial activity [43]. However, these reports contrast with others in which the variability of the properties of propolis collected in different months is reported, although this last work concerns propolis from Taiwan [44]. Additionally, it has been reported that a type of propolis from France has antibacterial activity against 20 different bacterial strains; in contrast, there is variability between the active concentrations of this propolis [37]. Moreover, Mexican propolis from different areas of the north has antimicrobial activity, in particular against *Staphylococcus aureus*, as well as a high antioxidant activity [39]. Another study reported the antioxidant activity of propolis from different countries: those from Argentina, Australia, China, Hungry, and New Zealand, of which the latter presents higher antioxidant activity, and this property is related to the content of polyphenols [31].

Propolis is known to stimulate the vascular endothelial growth factor and significantly intensifies cell proliferation, which is why it has been tested in conjunction with creams used in the treatment of skin wounds; this mixture speed up the healing process compared to standard treatment [45]. In line with this, propolis ethanolic extracts showed higher anti-inflammatory and healing effects on oral wounds [46,47]. Among the biological properties of propolis, the anti-inflammatory activity is widely studied [48,49]; this property has been reported in Korean propolis [50] and Brazilian propolis, which are also considered immunomodulators [48,51,52]. These biological activities are usually associated with the flavonoid content in this propolis, and these compounds have pharmacological value because they can prevent gastric ulcer formation by antioxidant and antisecretory mechanisms [8,53,54,55,56]. Due to the above, the aim of the present review was to evaluate the gastroprotective potential of propolis in the context of its function against NSAID-induced gastric ulcers.

## 2. Materials and Methods

A modification of the search process used by Silva et al. and Fazalda et al. was carried out [6,8]. PubMed (https://pubmed.ncbi.nlm.nih.gov (accessed on 29 March 2021)) and Science Direct (https://www.sciencedirect.com (accessed on 29 March 2021)) were used for the search process. The database criteria included the terms “propolis and gastric ulcer, propolis and gastro-protective, propolis and peptic ulcer disease, propolis and NSAID, propolis and NSAID-induced gastric ulcer”; moreover, only research articles with a publication date from 2000 to 2021 were taken into account.

As selection criteria, only articles published in the English language and studies with the following characteristics were taken into account: (1) original papers with full text, (2) using NSAIDs as one of the ulcer inducers in murine models, and (3) using propolis as treatment. Article exclusion criteria were: (1) review articles; (2) articles written in another language; (3) studies from news, letter, editorials, or social media; and (4) duplicated studies.

**Figure 1:**
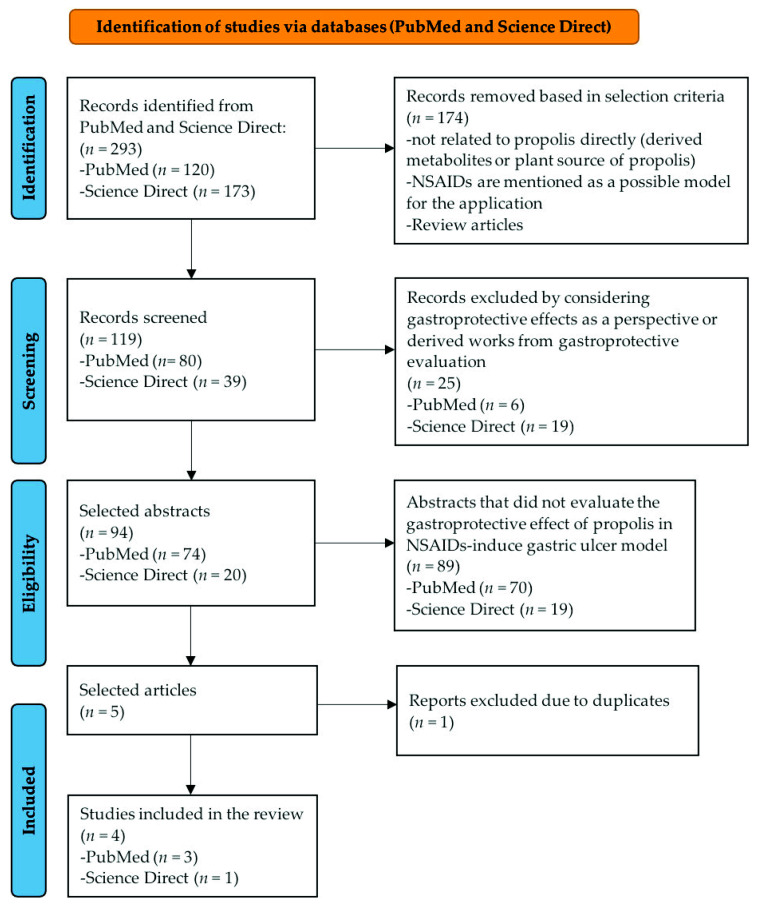
PRISMA flow diagram to show the literature selection process in PubMed and Science Direct databases used in this review.

**Figure 2:**
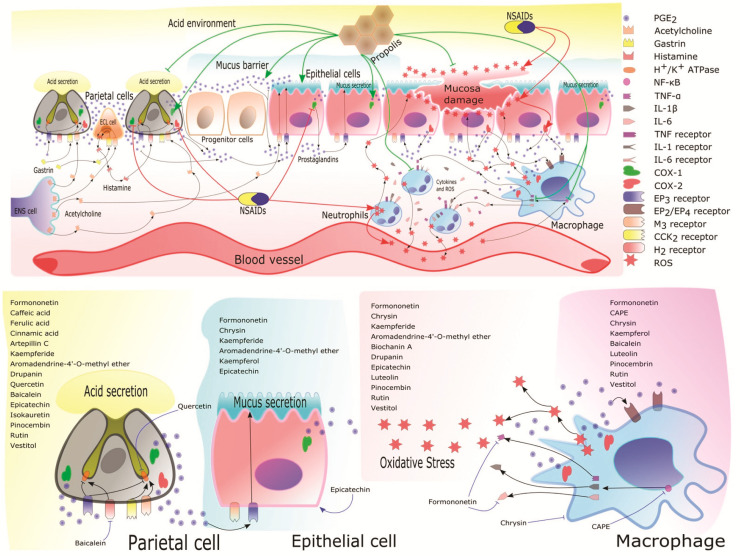
Physiological regulation of gastric mucosa and key factors involved in the gastroprotective effect of propolis against NSAIDs-induced gastric ulcers. Black arrows and slashes show the relationship between the cells that are present in gastric mucosa. The ganglion cell of the enteric nervous system (ENS) that secretes histamine interacts with parietal cells and epithelial cells to regulate the acid and mucus secretion, respectively; additionally, it induces the secretion of histamine in the enterochromaffin-like cells (ECL), and, together with the gastrin, induces acid secretion in parietal cells. COX isoenzymes (COX-1 and COX-2) and their localization in the gastric mucosa cells are presented, as well as the production of prostaglandin E_2_ (PGE_2_), which is a key molecule that regulates mucus production in epithelial cells and acid suppression in parietal cells; moreover, it is implicated in the pro-inflammatory response exerted by leucocytes as a response to the increase in the prostaglandin secretion by COX-2, as well as the reduction in blood flow in the gastric mucosa. The COX suppressor activity of NSAIDs and their adverse effects in the gastric mucosa cells, such as the capacity for generating COX-independent damage in epithelial cells by inducing reactive oxygen species (ROS) production, are shown by red arrows and slashes. On the other hand, green arrows and slashes show the key factors implicated in the gastroprotection of propolis in the gastric mucosa, as well as its antioxidant and anti-inflammatory effects in the gastric tissue. Finally, membrane receptors and their agonists are included in the figure according to their cellular localization and physiological function in the gastric mucosa. Additionally, are shown some secondary metabolites identified in propolis samples that display gastroprotective effects and the cell types in which they have their activity. In the yellow section are named secondary metabolites that have antacid activities; the blue section is listed secondary metabolites that stimulate gastric mucus secretion; the red section is shown secondary metabolites that own antioxidant activity, and the purple section is shown secondary metabolites that displays anti-inflammatory activities related to gastric ulcer disease. It should be noted that the blue arrows and slashes are shown specific activity of some secondary metabolites as the baicalein that display their suppressive activity of acid secretion in parietal cells by means of H2 receptors. Quercetin inhibits the proton pump ATPase activity of parietal cells. On the other hand, epicatechin has cytoprotective activity in epithelial cells of the gastric mucosa; and formononetin (an inhibitor of TNF-α and IL-6), chrysin (that is an immunomodulator), and CAPE (an inhibitor of NF-κB) display their activities on leukocytes of gastric mucosa in the ulcerative process.

**Figure 3:**
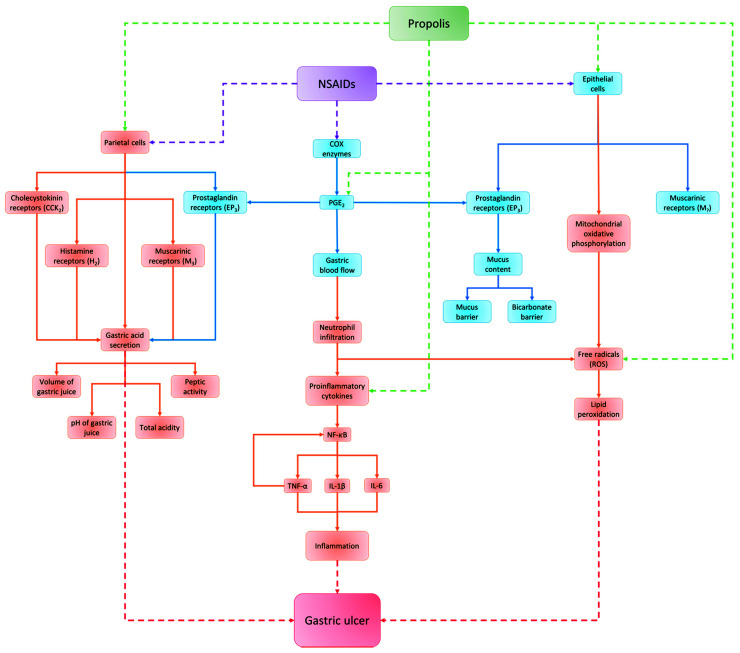
Harmful and protective factors implicated in the development of gastric ulcers. Blue boxes and arrows show the protective factors of the gastric mucosa, whereas orange boxes and arrows show harmful factors involved in the development of gastric ulcers. The purple dashed arrows and box show the level at which NSAIDs act to induce gastric ulcers in the gastric mucosa; on the other hand, the green dashed arrows and box show the different levels at which propolis can act to display its gastroprotective and antiulcerogenic properties. Finally, the red dashed arrows and box show the factors that lead to the development of gastric ulcers.

**Figure 4:**
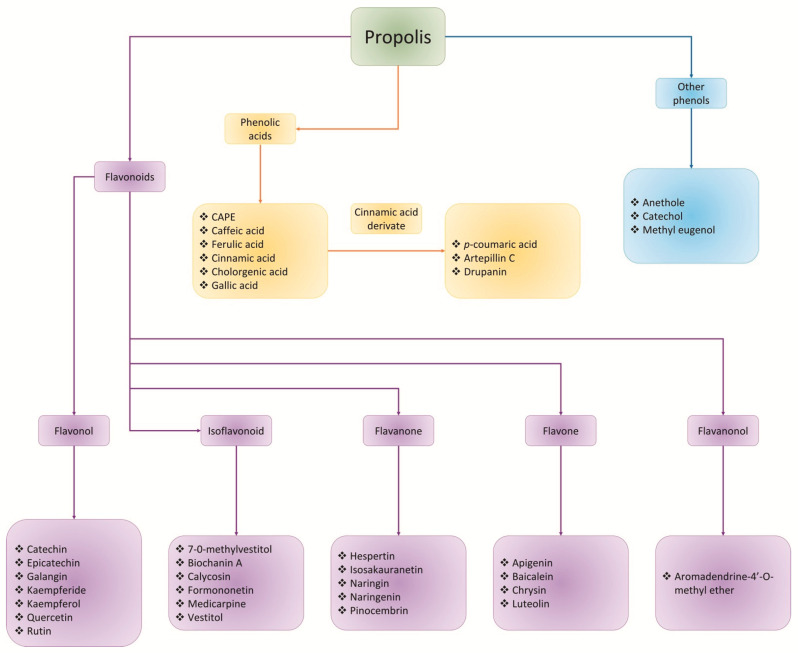
Class of phenolic compounds contained in propolis related to their gastroprotective activity. Purple boxes shown flavonoid types reported in the chemical composition of different propolis with gastroprotective activity. Yellow boxes are shown phenolic acids as well as cinnamic acid derivate compounds recorded in propolis samples. Blue boxes are shown other phenolic compounds reported with gastroprotective activity identified in propolis samples of Brazil and Mexico.

All tables and figures were designed and made by the authors of this review and the images used do not have copyright issues. Figure 1 was made with PowerPoint (16.43) Microsoft software; Figure 2 was made with the scalable vector graphics editor InKscape (1.0.2) and the mind maps (Figure 3 and Figure 4) were made with PowerPoint (16.43) Microsoft software. All figures and images were editing and escalated with the GNU image manipulation program GIMP (2.10.22).

## 3. Results

### 3.1. Selection and Study Characteristics

A total of 293 articles were identified according to the terms used in both databases revised in this review; from these, 120 articles were from PubMed and 173 were from Science Direct. According to the selection and exclusion criteria, only four articles complied with the selected characteristics for this work. The search process of articles selected and included in this work is summarized in the flow diagram of Figure 1. The selected articles were published between the years 2007 and 2021 [57,58,59,60] and were published in different journals, which were Journal of Ethnopharmacology [57]; International Journal of Radiation Biology [58]; *Nutrients* [59]; and Biomedicine and Pharmacotherapy [60]; all of these are indexed journals and have a journal impact factor (JIF) of 3.690, 2.368, 4.546, and 4.545 in 2019, respectively, according to InCities Journal Citation Reports (https://jcr.clarivate.com/JCRLandingPageAction.action (accessed on 29 March 2021)).

Table 1 shows a summary of the general characteristics of the studies selected for this review. In three of four works, the authors used Wistar rats with body weight in a range of 150–320 g [57,58,59]; two of them only worked with male rats [57,58] and only one worked with both rat sexes [59]; finally, the last worked with male ICR mice with a bodyweight of 25 ± 5 g [60] for the NSAID-induce gastric ulcer model. In all works, the NSAID selected to induce gastric ulceration was indomethacin, although this drug was given to the organism at different doses. Additionally, three studies administered indomethacin via the intragastric route, also named the oral route [57,59,60], and one study used indomethacin via the intraperitoneal route [58].

The indomethacin doses of the studies that administered the NSAID intragastrically were 100 mg/kg, and this work also evaluated propolis from different geographical regions of Brazil [57,59]; in another study, indomethacin was administered at a dose of 20 mg/kg, and the propolis evaluated was from the north of Mexico [60]. In the study that induced gastric ulceration via the intraperitoneal route, they used an indomethacin dose of 10 mg/kg, although they did not specify the origin of the propolis and mentioned that the propolis sample used was obtained from a Danish company that made an aqueous propolis extract manufactured from suppliers of different world regions [58]. As reference drugs, H_2_RAs (cimetidine at 100 mg/kg p.o.) were used, and in these studies, the gastroprotective activity of propolis was evaluated in other models in addition to indomethacin, such as ethanol and stress models [57,59] and PPIs (lansoprazole at 15 mg/kg p.o. and omeprazole at 20 mg/kg p.o.) [58,60].

The articles that evaluated the gastroprotective activity of Brazilian propolis used doses of 50, 250, and 500 mg/kg [57,59]; similarly, the Mexican propolis was evaluated in doses of 50, 150, and 300 mg/kg [60]; all of these used ethanolic extracts; in contrast, the article that used the Danish propolis only evaluated a dose of 650 mg/kg [58]. In all cases, the propolis sample was analyzed by means of HPLC analysis, and the parameters evaluated related to the gastroprotective effect of the samples, including the estimation of the ulceration lesion index, percentage of the lesion area, mucin or mucus content, and the determination of gastric secretion, which takes account the gastric content volume, pH, total acidity, and peptic activity. Moreover, some authors also evaluated the antioxidant capacity of the propolis sample, and some parameters of the antioxidant activity related to the ulcer process such as the content of malondialdehyde (MDA), glutathione (GSH), and superoxide dismutase (SOD) activity; they also measured the content of prostaglandin E_2_ (PGE_2_), tumor necrosis factor-alpha (TNF-α), interleukin-1-beta (IL-1β), interleukin-6 (IL-6), and myeloperoxidase (MPO).

### 3.2. Effect of the Propolis in Gastric Ulcer Healing

With respect to the gastroprotective activity of propolis, different studies show the capacity of this natural product to reduce gastric ulcers [57,58,59,60,61,62,63,64,65,66]; of these, a lower number of studies have been directed toward the activity of propolis against gastric ulcers induced by NSAIDs. Nevertheless, some authors have studied the activity of some regional propolis on NSAID-induced gastric models. In this way, green Brazilian propolis induced a significant decrease in gastric ulceration with a dose of 500 mg/kg in male rats that were treated with indomethacin (100 mg/kg) orally to induce gastric damage. This work also evaluates green propolis activity with other models of gastric ulcer induction, such as ethanol-induced gastric ulcers and water immersion stress-induced gastric ulcers; in both, green propolis showed gastroprotective activity in both ulceration models, although in lower doses than the NSAID model. Moreover, the authors showed the antisecretory activity that green propolis exhibited at doses of 250 and 500 mg/kg, which included the reduction in the gastric juice volume, total acidity, and pH (Table 1). Finally, the HPLC analysis showed the presence of phenolic compounds such caffeic acid, *p*-coumaric acid, and 3-prenyl-4-hydroxycinnamic acid (artepillin C), as well as the flavonoids isosakuranetin and aromadendrine-4′-methyl ether (Table 2) [57].

Similar to Brazilian green propolis, other studies have evaluated the gastroprotective potential of Brazilian red propolis [59,63], which, unlike green propolis that is produced in southeastern Brazil, is abundant in the northeast of this country [63]. Discussing specifically the antiulcer activity of this propolis on NSAID-induced gastric ulcer model, de Mendoça et al. [59] reported that the doses of 250 and 500 mg/kg of red propolis are effective in reducing the gastric ulceration produced by oral administration of indomethacin (100 mg/kg) with inhibition percentages of 87.34% and 100%, respectively. In this study, red propolis was analyzed by means of HPLC, and the authors reported that the major component of the propolis sample was the flavonoid formononetin, which, also presented the capacity to inhibit 100% of the ulceration induced by an indomethacin dose of 10 mg/kg. In this context, both red propolis doses and formononetin also presented gastroprotective activity in an ethanol-induced gastric model in a dose-dependent manner. This property is explained by the authors as due to the capacity of red propolis and its major constituent to reduce the gastric secretion volume and increase the mucus production in the stomach, as well as the antioxidant and the anti-*Helicobacter pylori* capacity of red propolis.

Propolis from a Danish company also displayed gastroprotective activity against indomethacin-induced gastric ulcers administered intraperitoneally (10 mg/kg) in rats exposed and non-exposed to ionizing radiation; therefore, the number of gastric lesions was decreased with the pre-treatment of propolis at a dose of 650 mg/kg. The above was associated with a reduction in acid output and peptic activity of this propolis in a similar manner to the Brazilian propolis samples evaluation. Moreover, Danish propolis increased the secretion of mucin, which is related to the capacity of this propolis to increase the levels of PGE_2_ in irradiated and non-irradiated rats; additionally, after the treatment with the propolis, the levels of TNF-α and IL-1β were suppressed, and the lipid peroxidation was also reduced (Table 1). This propolis sample also contains traces of different flavonoids, although the authors report the caffeic acid phenethyl ester (CAPE) as the only phenolic compound identified in their HPLC analysis (Table 2) [58].

A study exploring the key factors involved in the gastroprotective activity of propolis in the indomethacin-induced gastric ulcer model in mice was conducted by Ruiz-Hurtado et al. [60]. In this work, propolis from North Mexico was evaluated and compared with PPIs (omeprazole 20 mg/kg) with an oral pre-treatment of different doses of Mexican propolis (50, 150, and 300 mg/kg) (Table 1). The anti-ulcerative effect of this propolis was of a dose-dependent form, although the most active doses were 150 and 300 mg/kg, which reduced the gastric injuries by 89.96% and 96.70%, respectively, and was similar to the antiulcer effect of omeprazole (91.69%). Additionally, the histopathological analysis of the experimental groups was presented. The authors observed a reduction in different parameter indicators of gastric mucosa damage, such as the presence of congested blood vessels, mucosal hemorrhage, inflammatory cell infiltration, and mucosal edema. It is worth mentioning that de Mendoça et al. [59] also reported a histopathological analysis in their study, but this analysis was performed on the ethanol-induced gastric ulcer model and not on the NSAID model; therefore, we cannot observe the damages caused by the use of indomethacin at the histological level in this work.

The gastroprotection of Mexican propolis is demonstrated by means of the increase in PGE_2_ levels, which is related to the maintenance of the mucus content in the gastric mucosa, and the decrease in MPO, which is reflected in the reduction of neutrophil infiltration and has a direct effect on decreasing the levels of inflammatory markers TNF-α, IL-6, and IL-1β. On the other hand, this propolis also had an effect on the reduction of lipid peroxidation in the gastric tissue, associated with the increase in the activity of the antioxidant enzymes SOD and GPx caused by Mexican propolis. Moreover, the study also reported the antioxidant capacity of this propolis, related to the high content of phenolic compounds and flavonoids such as apigenin, baicalein, catechol, catechin, chrysin, kaempferol, naringin, naringenin, and pinocembrin (Table 2). It should be noted that this is the only study where the acute toxicity of propolis was evaluated, and this was classified as a natural product with low acute toxicity and belongs to category five according to the Globally Harmonized System of Classification and Labeling of Chemicals (GHS).

## 4. Discussion

NSAIDs are drugs known for their potential to inhibit inflammation in diverse disorders that affect human health. The anti-inflammatory effects of these drugs occur through the inhibition of COX enzymes; nevertheless, this action mechanism is responsible for exerting the ulcerative potential of NSAIDs in the stomach. Indomethacin is an NSAID drug that displays its benefic and ulcerogenic effects by means of the inhibition of the PGE_2_ synthesis of COX enzymes. Moreover, indomethacin is known for its high potential to induce gastric ulcers; hence, this medicine is commonly used in several protocols to evaluate gastroprotective agents in murine models [57,60].

PGE_2_ has a protective function in the gastric mucosa. This compound is key in the mechanism involved in the mediation of the adaptative immune response in the stomach due to the regulation that it exerts on the increase in mucosal resistance by stimulating mucus synthesis by the epithelial cells of the gastric mucosa; additionally, it is implicated in the regulation of blood flow in the gastric tissue, and, finally, it is related to the diminishing of harmful factors to the stomach such as gastric acid secretions [57,60]. Regulation of blood flow in the stomach is important because the injury caused by NSAIDs is characterized by its reduction as well as reduced bicarbonate and mucus secretion, and, therefore, an increase in acid diffusion and inhibition of gastric mucosa repair [59]. Reduction in the blood flow of gastric mucosa promotes leukocyte and neutrophil infiltration in the tissue and is directly related to indomethacin-induced gastropathy; moreover, indomethacin can promote the adherence of neutrophils in the gastric endothelium by a COX-independent mechanism. This drug occludes blood microvessels and hence reduces the gastric mucosal blood flow [60,174] (Figure 2).

Neutrophil infiltration in the gastric tissue catalyzes the production of hypochlorous acid from H_2_O_2_ and the oxidation of tyrosine to tyrosyl radicals by means of H_2_O_2_ as an oxidizing agent; these stimulate the inflammation and induce apoptosis in the gastric mucosa [60]. Any harmful event that interacts with the gastric tissue is perceived by the macrophages and monocytes in the stomach as noxious effects of NSAIDs in the epithelial gastric mucosa cells. These immune cells can secret cytokines as TNF-α, IL-1β, IL-6, and NF-κB. These molecules play a key role by translocating to the nucleus of gastric epithelial cells and promoting the expression of many target genes such as pro-inflammatory cytokines mentioned above [175,176,177].

Pro-inflammatory genes, in addition to initiating injury in the gastric mucosa, also have a role in a positive feedback loop that can amplify the damage in the gastric mucosa. TNF-α is a cytokine that promotes inflammation and by itself is an efficient activator of NF-κB, so this cytokine promotes the destruction of gastric tissue; moreover, it is involved in the recruitment of neutrophils and other leucocytes by means of the induction of adhesion molecules in these cells. In addition, TNF-α is related to the accumulation and production of superoxide molecules as a consequence of the accumulation and activation of neutrophils in the stomach tissue that produces disturbances in the microcirculation and thus the production of free radicals [58,60,175,176,177,178]. Nevertheless, PGE_2_ can inhibit TNF-α production [179]; therefore, the modulation of pro-inflammatory cytokines is directly related to the diminished ulcerogenic effects of NSAIDs.

In this context, propolis has the capacity of reducing the lymphocytic infiltration in the gastric tissue. Brazilian, Danish, and Mexican propolis showed promising results. Brazilian and Mexican propolis evidenced their capacity to decrease neutrophil infiltration at the histological level as well as the level of pro-inflammatory cytokines, such as Mexican and Danish propolis; in both cases, the propolis reduced the levels of the inflammatory cytokines TNF-α and IL-1β independent of the administration route that was used in these studies (oral and intraperitoneal, respectively). Moreover, the Mexican propolis also reduced the level of IL-6 and also decreased the concentration of MPO in the gastric tissue, which is an enzyme secreted and localized in activated neutrophils and is thus associated with the infiltration of neutrophils in the tissues [58,60].

In the case of Brazilian red propolis [59], the phytoestrogen formononetin, an O-methylated isoflavone present in the chemical composition of this propolis, had the capacity to reduce neutrophil infiltration, which suggests an immunomodulatory effect in the release of cytokines in the gastric mucosa. The evidence for this is the study of a geopropolis collected in the same region as Brazilian red propolis, which was shown to also reduce neutrophil infiltration [180]; the formononetin activity was related to its capacity to inhibit TNF-α and IL-6 expression and improve SOD activity in traumatic brain injury [127]; thus, this phytoestrogen mediates, in part, the gastroprotective activity of red propolis.

CAPE, a component of green and Danish propolis [57,58], has been reported to have a potent and specific inhibition of NF-κB activation; additionally, it is known for its anti-inflammatory and antioxidant properties [181]. Finally, Mexican propolis also reduced the level of pro-inflammatory cytokines TNF-α, IL-1β, IL-6, and, moreover, chrysin was present in its chemical composition [60]. This flavonoid had gastroprotective activity at doses of 50 to 100 mg/kg in NSAIDs-induced gastric ulcer models by means of the phenotypic differentiation of pro-inflammatory M1 macrophages to anti-inflammatory M2 macrophages. This process is carried out through the activation and upregulation of peroxisome proliferator-activated γ (PPAR-γ) expression; additionally, chrysin increases the mucus secretion, helps in the repair of gastric mucosa by means of the upregulation of angiogenesis, and promotes the antioxidant enzyme activity [56].

Pro-oxidant effects of indomethacin are well known; the production of free radicals such as reactive oxygen species (ROS) can occur through neutrophil infiltration, as mentioned above. These cells can produce ROS (Figure 2); thus, the reduction in the infiltration of neutrophils can reduce the ROS formation in gastric mucosa by 50% [166]. Furthermore, indomethacin can inhibit mitochondrial oxidative phosphorylation in the gastric mucosa epithelial cells. This releases the cytochrome c from the mitochondrial intermembranous space into the cytosol and finally releases inner ROS in the cells. The release of Ca^+^ from the mitochondria, the reduction in the intracellular ATP concentration, and the cellular imbalance in osmotic and lipid peroxidation are produced by the ROS in the epithelial cells. The sum of the noxious effects of ROS increases the permeability of the epithelial gastric cells and finally produces mucosal damage in the stomach [175].

In general, propolis is known for its antioxidant effects; several studies show the capacity of propolis to decrease oxidative stress in different models inducing gastric ulcers [58,59,60,61,62,63,65,66,182,183]. The antioxidant effects also are involved in the gastroprotection of propolis in NSAIDs-induced gastric ulcers [58,59,60] (Figure 3). These studies have in common the reduction in lipid peroxidation of propolis in the gastric tissue. This is explained by the increase in the activity of the antioxidant enzymes SOD, CAT, and GPx, which is promoted by the oral treatment with propolis [60,62,63]. Antioxidant enzymes are important due to the SOD enzyme catalyzing the dismutation of superoxide radical (O_2_^−^) to hydrogen peroxide (H_2_O_2_); this molecule is captured by the GPx and CAT enzymes and transforms H_2_O_2_ into H_2_O and O_2_ [175].

Moreover, the propolis studied in NSAID-induced gastric ulcers is rich in phenolic compounds such as coumarins, tannins, procyanidins, xanthenes, and, in particular, flavonoids, which, in their chemical composition, have the capacity to scavenge ROS by means of donating their hydrogen atoms from their hydroxyl groups [166,179]. Flavonoids also are known to chelate transitional metal ions such as iron, and this effect could deprive ROS of an important element involved in their effect on lipid peroxidation [58].

There are many compounds identified in propolis samples that have gastroprotective activity in NSAIDs-induced gastric ulcer models, principally from Brazilian (green and red) propolis, and North Mexican propolis, as shown in Table 2; among all these phenolic compounds, some have been reported with gastroprotective effects such as caffeic acid, ferulic acid, cinnamic acid, *p*-coumaric acid, artepillin C, Kaempferide, 7-O-methylvestitol, aromadendrine-4′-O-methyl ether, formononetin, biochanin A, medicarpine, apigenin, catechin, kaempferol, naringin, and naringenin [53,54,59,63,68,70,76,88,92,93,94,95,96,98,126,127,128,153,154] or activities related to gastroprotective effects; between them, most classes of phenolic compounds related to the gastroprotective activity of propolis are flavonoids, that include principally five subclasses of this type of compounds (flavonols, isoflavonoids, flavanones, flavones, and a flavanonol) and, phenolic acids, among them compounds derivate of cinnamic acid; and finally, some simple phenols that have been reported with gastroprotective activity as the anethole, catechol and methyl eugenol (Figure 4). Nevertheless, the gastroprotective-related activities of all the phenolic compounds in these propolis samples have not been evaluated. Therefore, each compound represents an opportunity area for the development of drugs for the treatment of gastric ulcers, excluding the compounds not identified in these propolis samples with activity against NSAID-induced gastric ulcers.

Biochanin A, a flavonoid found in Brazilian red propolis, has gastroprotective effects by means of the strong induction of SOD and nitric oxide enzymes, which is reflected in the reduction in lipid peroxidation [59]. Artepillin C is a major compound of Brazilian green propolis, although drupanin (3-prenyl-4-hydroxycinnamic acid) and the flavonoids aromadendrin-4′-O-methyl-ether and kaempferide also are present. All these compounds display gastroprotective activities on indomethacin-induced gastric ulcers through the normalization of the SOD enzyme and the reduction of lipid peroxidation [62,68]. In a similar form, flavonoids content in Mexican propolis, kaempferol, apigenin, catechin, naringin, and naringenin showed gastroprotective effects [60]. For example, kaempferol reduced the pro-inflammatory response in damaged gastric tissue, increased the NO production, and preserved the gastric mucus glycoprotein [53,54].

Flavonoids found in propolis can stimulate and increase mucus and bicarbonate production by means of stimulation of PGE_2_ in the gastric epithelial cells and can affect the proton pump activities in gastric parietal cells [58,59]. Formononetin and 7-O-methylvestitol from Brazilian red propolis and aromadendrin-4′-O-methyl-ether and kaempferide from Brazilian green propolis increased the mucin content in damaged gastric tissue [59,63,68]. Thus, mucus content contributes to the gastroprotective effect of propolis because the mucus and bicarbonate barrier provides protection to block the diffusion of ROS to the inner part of gastric epithelial cells [178,184] (Figure 2). 

Other important properties that flavonoids present are their cytoprotective and antisecretory effects in gastric damage models. The HPLC analysis of Brazilian green propolis revealed the presence of prenylated cinnamic acid derivates and flavonoids [57]. Moreover, a posterior study of the phenolic compounds (caffeic, ferulic *p*-coumaric, and cinnamic acids) of green propolis showed the anti-ulcerative activity of these compounds in an NSAID-induced gastric ulcer model, and the possible mechanism of action of phenolic compounds derived from green propolis is through their inhibition of antisecretory effects because the three compounds reduced the volume of gastric juice and total acidity and increased the gastric pH at doses between 50 and 250 mg/kg. Additionally, none of the compounds studied presented acute toxicity at doses higher than 2000 mg/kg [98]. On the other hand, the isolated compounds from this propolis such as artepillin C, drupanin, aromadendrin-4′-O-methyl-ether, and kaempferide presented antisecretory effects, and, in a similar form to that of the phenolic acids named previously, these four phenolic compounds reduced the volume of gastric juice and total acidity; however, unlike phenolic acids, these compounds reduced the pH and pepsin activity of the gastric juices [68].

The study elucidated the antiulcerogenic activity of Brazilian red propolis as well as the formononetin isolated from this and showed a reduction in gastric secretion volume in a pylorus-ligated model [59]. Is important to note that both treatments were administered via the intraduodenal route for the evaluation of their antisecretory effects; thus, the systemic effects of both treatments are probably not related to the neutralization of gastric acid. This is coupled with the fact that both treatments did not reduce the total acidity and pH, indicating that the antisecretory effects of the treatments do not play an important role in their gastroprotective activity. These results are consistent with a recent study that evaluates the gastroprotective activity of Brazilian red propolis in a model of gastric ulceration by means of oral administration of ethanol acid, where the red propolis did not display antisecretory action [63].

Gastric acid secretion is relevant in the formation of ulcers in the gastric tissue by the ingestion of NSAIDs [174] (Figure 3). In this context, El-Ghazaly et al. [58] studied the gastroprotective activity of a propolis sample from Denmark in rats irradiated and non-irradiated with gastric damage induced by indomethacin. In this work, a pivotal role was the antisecretory action of propolis in both experimental groups due to the stomach being a radiosensitive organ that does not tolerate the radiation doses used in radiotherapy for the control of cancer. Therefore, when the stomach is exposed to radiation, it can produce ulcers, perforation, chronic atrophic gastritis, and alteration of secretory and motor functions. These alterations in the stomach are associated with the increase in gastric acid secretions, which were reduced with the treatment of propolis. This effect of propolis is probably associated with its content of flavonoids, such as quercetin, which inhibits the proton pump-ATPase activity of parietal cells and suppresses gastric acid secretion [58,185,186]. Another example is baicalein, which reduces the inflammatory process, stimulates the antioxidant system, inhibits gastric secretion via the histaminergic pathway, and mediates the proton pump-ATPase activity [55].

Several studies have indicated the beneficial activities of propolis around the world in human health, such as the antimicrobial, antioxidant, wound healing, antitumoral, and anti-ulcerative properties of this natural product. The safety commonly associated with the use of propolis and other products used in folk medicine usually gives us a reference of the possible applications for their evaluation and posterior use in the treatment of diseases that affect human health. In this review, propolis is presented as an alternative for the treatment of gastric disorders such as gastric ulcer disease, which is a global issue, and its development is associated with the use of NSAIDs both in hospital and non-hospital settings where their use for the treatment of inflammatory disorders can lead to the development of gastric disorders either as an inherent side effect of these drugs or associated with gastric infections with *H. pylori*, which aggravate the ulcerative process in the gastric mucosa of the stomach [2,4,8].

The increase of PGE_2_ level associated with the gastroprotective effect of propolis could give us information about the antagonistic effect of secondary metabolites contained in propolis with NSAIDs because their anti-inflammatory activity is related to the inhibition of COXs enzymes and, therefore, could leading the increase of dosage with NSAIDs to get the same anti-inflammatory properties; nevertheless, anti-inflammatory effects of phenolic acids and flavonoids of propolis could help in the decrease of the inflammatory process and help in the therapy with NSAIDs. These compounds display their activity by the inhibition of transcription factors, decrease of cytokines and chemokines, as well as concomitantly pathways to the COXs inhibition [49,50,88,120]. In line with this, anti-inflammatory effects of apigenin are related to the inhibition of inducible nitric oxide synthase (iNOS), COX-2 enzyme, and cytokines as IL-1β, IL-6, and TNF-α [88,89]. Moreover, the decrease of inflammation of naringenin is related to the inhibition of oxidative stress, MPO activity, leukocyte recruitment, NF-κB, and the cytokines IL-1β, IL-6, TNF-α and IFN-γ [120,152]. And chrysin modulates macrophage differentiation of pro-inflammatory M1-sate to anti-inflammatory M2-state [56].

Taking the above into account, this review is highly important for identifying the effectiveness of propolis as a gastroprotective and anti-ulcerative agent for the treatment of gastric ulcers induced by the consumption of NSAIDs, as well as the range of effective propolis doses that display positive antiulcer activities for their evaluation in murine models and the key factors involved in their gastroprotective activity. Likewise, with the aim of understanding the comparison of the different studies carried out on the properties of propolis in gastric wounds associated with the consumption of NSAIDs (Figure 2 and Figure 3), only articles that used this type of drug as a medium to induce gastric ulcers in mice models were taken into account to compare the reduction in the length of the stomach wounds and the cytoprotective effects of propolis directly related to the protection that is displayed in the ulcerogenic process. Nevertheless, the selection and exclusion criteria used in the present review excluded two works that evaluated the activity of African propolis on NSAID-induced gastric ulcer murine models due to these works not being included in the databases used for the search process of this study, and, additionally, these works were published in non-indexed journals without an impact factor.

A limitation of the research on propolis is the fact that there is high variability in the chemical composition as the geographical zone, plant source, collection season, bee species, and solvents used in the extraction directly influence the chemical components found in propolis. All these factors make it difficult to standardize propolis both in material and analytical methods [8,35,60]. In most of the articles used in this review, the geographic collection area was established; nevertheless, one study did not give precise information on the area where the propolis was obtained when opting for commercial propolis [58]. Different propolis doses were studied in a range from 50 to 650 mg/kg, and the different experimental groups used, and the evaluation of some parameters was not present in other articles; additionally, the differences between indomethacin doses and murine fasting for the evaluation of the activity of propolis on gastric ulcers are noted in this work [57,58,59,60].

The need to establish the mechanism involved in the antiulcer activity of propolis is evident. The studies that evaluate the protection that propolis displays in damage processes in gastric mucosa should standardize the doses and experimental groups used for the evaluation of the different propolis and take into account the biological parameters to be evaluated, as well as standardizing the NSAIDs dose used for the induction of gastric ulcers in the stomach on murine models and, finally, whenever possible, give the precise origin of the commercial propolis evaluated in order to identify the differences or similarities of the propolis used in particular studies. The above is due to the complexity in the chemical composition of propolis because the variation in its composition modulates and modifies its effects; this can appear to be a virtue of propolis because the unique properties and activities of each propolis from diverse countries are a source of compounds that represent new alternatives for the treatment of diverse diseases worldwide.

Clinical applications of propolis for the treatment of gastric ulcer disease should be considered. Although currently there is little evidence about the gastroprotective activity of propolis inpatients. Coelho et al. [187] reported the effect of propolis to eliminates *H. pylori* infection in patient voluntaries; nevertheless, this natural product had minimal antibacterial effect on the infected patients, although, in the same work the authors consider the necessity of realizing a larger study that includes modifications in the administration and doses evaluates, and a longer duration of the treatment with propolis as well as increase the number of patients to define the activity of propolis on *H. pylori*.

On the other hand, there are clinical studies that report the antioxidant effects of this bee product, such as the work of Jasprica et al. [188] who reported the antioxidant effect of propolis given daily by 30 days to healthy women and men. In this work, authors denote that the effect of propolis seems to be related to time and gender due to the male population treated with propolis reduce their lipid peroxidation and increase the SOD enzyme activity; whereas the female population evaluated did not present any change associated with the treatment with propolis in the antioxidant parameters evaluated. Another study, reports the antioxidant activity of a standardized extract of propolis in healthy people, in which, the extract increase antioxidant enzymatic capacity significantly, in special, propolis increase the activity of the SOD enzyme in this patients, although authors there is not reported a correlation of this antioxidant activity of propolis with the sex of patients [189].

Moreover, the anti-ulcer activity of propolis has been evaluated in human trials; Henshaw et al. [190] reports the anti-ulcer activity of propolis on patients that present diabetic foot ulcers. In this study, patients show a well-tolerated reaction with the treatment of propolis and enhance wound closure when the weekly topical propolis was applied. In addition, Samet et al. [191] showed that propolis is effective to reduce the number and recurrence of aphthous stomatitis in patients affected. In this work, the authors propose that treatment with propolis could confer some advantage with respect to the patients that do not respond to other treatments for this illness. Additionally, quercetin a flavonoid usually present in the chemical composition of propolis (view Table 2) has been reported with the capacity to enhancing the healing of the aphthous ulceration process in a randomized clinical trial [192]. All these studies give us clues about the clinical approach of propolis in human health; therefore, clinical investigations are needed to determine if the propolis gastroprotective activity reported in animal models can be taken advantage of in human health.

## 5. Conclusions

Propolis has a higher potential as a gastroprotective agent for the treatment of NSAIDs-induced gastric ulcers. This fact is supported by many investigations that take propolis as a source of gastric protection. The protection that propolis provides to the mucosa occurs through the suppression of the release of noxious factors, such as gastric acid secretions and pro-inflammatory cytokines and by its antioxidative, anti-inflammatory, and cytoprotective effects. However, research on the compounds responsible for the gastroprotective activity of propolis is required to determine their potential use.

## Figures and Tables

**Table 1:** Characteristics of each study that complied with the selected characteristics for this work.

Work	Model-Induced Ulcer and Dose	Animal Model and Characteristics	Experimental Groups and Route of Administration	Methodology	Results
de Barros, M.P. et al., 2007 [57]	Ethanol 99.5% (1 mL) Indomethacin (100 mg/kg) Stress (immersed in water at 25 °C for 17 h)	Male Wistar rats, weighing 200–250 g with 12 h of fasting	Five groups (*n* = 6): 1 Vehicle (1% Tween-80 aqueous solution) 2 Cimetidine (100 mg/kg) or Omeprazole (30 mg/kg) 3 Green propolis (50 mg/kg) 4 Green propolis (250 mg/kg) 5 Green propolis (500 mg/kg)	Ethanol-induced gastric ulcer: After 12 h of fasting, the experimental groups were treated orally according to their group. One hour after, all groups received 1 mL of 99.5% of ethanol to induce gastric ulcers. After 1 h, animals were sacrificed by cervical dislocation, and the stomachs were removed and opened along the greater curvature. NSAID-induced gastric ulcer: After 12 h of fasting, the experimental groups were treated orally according to their group. One hour after, all groups received indomethacin (100 mg/kg p.o.) to induce gastric ulcers. After 4 h, animals were sacrificed by cervical dislocation, and the stomachs were removed and opened along the greater curvature. Stress-induced gastric ulcer: Experimental groups were treated orally according to their group. After 30 min, animals were placed in tubes and immersed vertically until the water reached the neck region in a tank with water at 25 °C for 17 h. After this period, animals were sacrificed by cervical dislocation, and the stomachs were removed and opened along the greater curvature.	In the ethanol-induced gastric ulcer, the three doses of green propolis and omeprazole reduced the lesion index, the total lesion area, and the percentage of the lesion in comparison with the vehicle group. In contrast, only the dose of 500 mg/kg of green propolis and the cimetidine group reduced gastric ulceration in the NSAID-induced gastric ulcer model. Whereas, in the stress-induced gastric ulcer, both the 200 mg/kg and 500 mg/kg green propolis doses reduced gastric ulceration.
El-Ghazaly, M.A. et al., 2011 [58]	Radiation exposure 6 Gy (0.48 Gy/min) and indomethacin (10 mg/kg)	Male Wistar rats, weighing 150–200 g with 48 h of fasting	Four groups (*n* = 8): 1 Vehicle (Tween-80) 2 Indomethacin (10 mg/kg) 3 Propolis (650 mg/kg) 4 Lansoprazole (15 mg/kg)	Assessment of the effect of propolis against gastric ulcers in normal animals: After 48 h of fasting, animals were administered with propolis or lansoprazole. After 1 h, animals were submitted to pyloric ligation surgery. After that, all experimental groups were given indomethacin (10 mg/kg i.p) with the exception of the vehicle group. After 4 h, the animals were anesthetized with ether and sacrificed by decapitation, trunk blood was collected, and the stomachs were removed and opened along the greater curvature. Assessment of the effect of irradiation and treatment with propolis on gastric ulceration: Different groups of rats were randomly allocated to receive a single radiation dose with a level of 6 Gy 24 h before indomethacin injection. After radiation dose, animals were administered with propolis or lansoprazole. After 1 h, animals were submitted to pyloric ligation surgery. At the end of the surgery, all experimental groups were given indomethacin (10 mg/kg i.p) with exception of vehicle group. After 4 h, the animals were anesthetized with ether and sacrificed by decapitation, trunk blood was collected, and the stomachs were removed and opened along the greater curvature.	Effect of propolis against gastric ulcer in normal animals: The treatment with propolis and lansoprazole reduced gastric ulceration by 75% and 87%, respectively. Both treatments reduced the free acidity and acid output and increased the gastric mucin content compared to the indomethacin group. Moreover, propolis and omeprazole protected against the reduction in PGE2 content and protected against the increase in inflammatory TNF-α and IL-1β mediators. Finally, both treatments reduced the lipid peroxidation to the normal values. Effect of irradiation and treatment with propolis on gastric ulceration: Exposure of animals to radiation before indomethacin injection increased gastric acidity and acid output significantly. Nevertheless, propolis and lansoprazole treatment had similar action on the parameters measured in the previous experiment.
de Mendonça, M.A. et al., 2020 [59]	Ethanol 99.5% (1 mL) Indomethacin (100 mg/kg)	Male and female Wistar rats, weighing 280–320 g with 24 h of fasting	Six groups (*n* = 6): 1 Vehicle (1% Tween-80 aqueous solution) 2 Cimetidine (100 mg/kg) or Omeprazole (100 mg/kg) 3 Red propolis (50 mg/kg) 4 Red propolis (250 mg/kg) 5 Red propolis (500 mg/kg) 6 Formononetin (10 mg/kg)	Ethanol-induced gastric ulcer: After 24 h of fasting, the experimental groups were treated orally according to their group. One hour after, all groups received 1 mL of 99.5% of ethanol to induce gastric ulcers. After 30 min, animals were sacrificed by cervical dislocation, and the stomachs were removed and opened along the greater curvature. NSAID-induced gastric ulcer: After 24 h of fasting, the experimental groups were treated orally according to their group. After 30 min, all groups received indomethacin (100 mg/kg p.o.) to induce gastric ulcers. After 6 h, animals were sacrificed by cervical dislocation, and the stomachs were removed and opened along the greater curvature.	Pre-treatments with the doses of red propolis inhibited the total lesion areas in a dose-dependent way; formononetin and omeprazole also reduced the area of total lesion significantly in the ethanol-induced gastric ulcer model. Moreover, histopathological damages induced by indomethacin in the gastric tissue were reduced with the treatments with red propolis. In the NSAID-induced gastric model, pre-treatments with the three doses of red propolis, formononetin, and cimetidine reduced the ulcer index with respect to the vehicle group; moreover, the 50 and 250 mg/kg doses of red propolis and formononetin reduced the secretion volume of gastric content; however, both treatments did not reduce the pH of gastric content. In contrast, the dose of 500 mg/kg of red propolis and formononetin increased mucus production in the stomach compared to the vehicle group.
Ruiz-Hurtado, P.A. et al., 2021 [60]	Indomethacin (20 mg/kg)	ICR mice weighing 25 ± 5 g with 12 h of fasting	Six groups (*n* = 6): 1 Vehicle (5% of sodium bicarbonate aqueous solution) 2 Indomethacin (20 mg/kg) 3 Omeprazole (20 mg/kg) 4 Chihuahua propolis (50 mg/kg) 5 Chihuahua propolis (150 mg/kg) 6 Chihuahua propolis (300 mg/kg)	NSAID-induced gastric ulcer: After 12 h of fasting, the experimental groups were treated orally according to their group. After 2 h, all groups received indomethacin (100 mg/kg p.o.) to induce gastric ulcers. After 6 h, animals were sacrificed by cervical dislocation, and the stomachs were removed and opened along the greater curvature.	The vehicle group did not develop gastric mucosal lesions, in contrast to the indomethacin group. Different doses of Chihuahua propolis and the treatment with omeprazole significantly decreased gastric injuries both macroscopically and histologically. Additionally, these treatments increased the mucus content in the gastric tissue and reduced lipid peroxidation. In line with this, the 150 mg/kg dose of Chihuahua propolis increased the SOD activity, and the 300 mg/kg dose increased GPx activity. On the other hand, the 150 and 300 mg/kg doses of Chihuahua propolis increased the PGE2 content, and both doses reduced the concentration of pro-inflammatory markers (TNF-α, IL-1β, and IL-6) as well as MPO content.

**Table 2:** Phenolic compounds identified in propolis with gastroprotective effect induced by NSAIDs.

Compound	Activity Related to Gastroprotective Effect							Country of Origin of Propolis Sample	Activity References
	Ga	Au	As	Af	Im	Ax	Cp		
2,2-dimethyl-6-carboxyethenyl-2H-1-benzopirane								Brazil [67]	
3-prenyl-4-dihydroxycinnamoiloxy cinnamic acid (baccharin)								Brazil [67,68]	
3-prenyl-4-hidroxycinamic acid (drupanin)	X	X	X	X		X		Brazil [57,68]	[68,69]
3,4-dimethoxycinnamic acid						X		Mexico [70]	[70]
3,5-diprenyl-4-hidroxycinamic acid (artepillin C)	X	X	X	X	X	X		Brazil [57,67,68,71,72,73]	[68,74,75]
3.3-dimethylallyl caffeate						X		Mexico [70]	[70]
5-methylchrysin ether						X		Mexico [76]	[76]
5-methylgalangin ether						X		Mexico [76,77]	[76]
5-methylpinobanksin ether						X		Mexico [76,77]	[76]
7-O-methylvestitol	X				X	X	X	Brazil [63]	[63]
Acacetin				X	X	X	X	Mexico, Brazil [78,79,80]	[81,82]
Alnusin								Brazil [83]	
Alpinetin				X		X	X	Mexico [76,77]	[76,84]
Alpinone					X			Mexico [77]	[85]
Anethole	X			X		X		Brazil [83]	[86,87]
Anisaldehyde								Brazil [83]	
Apigenin	X		X		X	X	X	Mexico [60,79]	[88,89]
Aromadendrine-4′-O-metyl ether	X		X					Brazil [57,68]	[68]
Baicalein	X	X	X	X	X	X	X	Mexico [60]	[55]
Benzoic acid				X		X		Brazil [71,83]	[90]
Biochanin A	X	X	X	X	X	X	X	Brazil [83,91]	[92,93,94,95,96]
Cadinene						X		Brazil [83]	[97]
Caffeic acid	X	X	X	X	X	X	X	Brazil, Mexico [57,70,72,73,76,98,99]	[76,98,100,101,102,103]
Caffeic acid phenethyl ester (CAPE)	X	X	X	X	X	X	X	Denmark, Mexico, Brazil [58,73,79,104]	[105,106,107,108,109]
Calycosin	X					X		Brazil [80]	[110]
Catechin	X			X		X		Mexico, Brazil [60,99,111]	[88,112,113]
Catechol	X	X		X		X		Mexico [60]	[114,115]
Cholorgenic acid	X	X	X	X		X	X	Brazil [73]	[116]
Chrysin	X		X		X	X		Mexico, Brazil [60,76,77,78,79,80,104,117]	[56,76]
Cinnamic acid	X	X	X	X	X	X	X	Brazil, Mexico [70,71,79,98]	[98,118,119]
Daidzein				X		X		Brazil [91]	[120,121]
Dalbergin								Brazil [80]	
Dihydrocinnamic acid								Brazil [71]	
Dihydroxy-methoxy chalcone								Brazil [122]	
Dihydroxy-methoxy flavanone								Brazil [122]	
Dihydroxy-trimethoxyflavone								Brazil [122]	
Dillenetin						X		Mexico [76]	[76]
Dimethoxy-dihydrochalcone								Brazil [122]	
Elemicin								Brazil [83]	
Epicatechin	X							Brazil [99]	[123]
Epoxypinocembrin chalcone						X		Mexico [70]	[70]
Ferulic acid	X	X	X	X	X	X	X	Brazil, Mexico [76,79,98,111]	[76,98,103,124,125]
Formononetin	X	X		X	X	X		Brazil [59,83,91,99]	[59,95,126,127,128]
Galangin	X		X		X	X		Mexico [76,79,117]	[76,129,130]
Gallic acid	X	X		X		X	X	Mexico, Brazil [72,79,99]	[131]
Genkwanin				X		X		Brazil [80]	[132]
Guaiacol				X		X		Brazil [83]	[133,134]
Hesperetin	X	X		X		X	X	Mexico [104]	[135,136,137]
Hispidulin				X		X		Brazil [80]	[110]
Isocholorgenic acid A				X	X	X	X	Brazil [73]	[138]
Isocholorgenic acid B								Brazil [73]	
Isocholorgenic acid C								Brazil [73]	
Isoelemicin								Brazil [83]	
Isopent-3-enyl caffeate						X		Mexico [70]	[70]
Isorhamnetin				X		X	X	Mexico [76,77]	[76,139]
Isorhamnetin-3-O-glucosylgallate								Brazil [122]	
Isosakuranetin (ponciretin)		X						Brazil [57]	[140]
Izalpinin						X		Mexico [70]	[70]
Kaempferide	X		X	X				Brazil, Mexico [68,77]	[68]
Kaempferol	X		X	X		X		Mexico, Brazil [60,70,78,79,99,122]	[53,54,70,88,120]
Liquiritigenin				X	X	X	X	Brazil [83]	[141,142,143]
Luteolin	X			X	X	X	X	Mexico, Brazil [78,79,111]	[89,112,135,144,145]
Medicarpin	X			X	X	X		Brazil [63,80,83]	[63,146]
Methoxy-dihydrochalcone								Brazil [122]	
Methoxyeugenol						X		Brazil [83]	[147]
Methyl eugenol	X			X		X	X	Brazil [83]	[148,149]
Myricetin-3-O-rhamnoside				X		X		Brazil [122]	[150,151]
Naringenin	X			X	X	X	X	Mexico [60,78,79,104]	[88,120,152]
Naringin	X	X		X		X		Mexico [60,78]	[88,153,154]
Neovestitol				X		X		Brazil [63,155]	[155,156]
Oblongifolin B								Brazil [63,80]	
*p*-coumaric acid	X	X		X	X	X		Brazil, Mexico [57,71,72,79,98,99]	[98,103,157,158]
Pentahydroxy-flavone-malonyl gallate								Brazil [122]	
Pinobanksin						X		Mexico, Brazil [77,80]	[159]
Pinobanksin-3-O-acetate								Mexico [79,117]	
Pinocembrin	X	X	X	X	X	X	X	Mexico, Brazil [60,70,72,76,77,78,79,91,104,117]	[70,76,77,85,129,160]
Pinostrobin						X		Mexico [70,77,79]	[161]
Prenyl-*p*-coumaric	X		X					Brazil [71]	[68]
Prenyl-pentahydroxy-flavone								Brazil [122]	
Quercetin	X	X	X	X	X	X	X	Mexico, Brazil [78,79,99,122]	[89,120,162,163,164]
Retusapurpurin								Brazil [83]	
Rhamnetin				X		X		Mexico [70]	[165]
Rutin	X	X	X	X		X	X	Mexico, Brazil [99,104]	[166,167]
Syringic acid				X		X	X	Mexico [76]	[76,168]
Tetrahydroxy flavonon								Brazil [71]	
Trans-ferulic acid				X		X		Brazil [99]	[169,170]
Tricin				X		X		Brazil [80]	[171,172]
Trihydroxy-dihydrocinnamic acid								Brazil [122]	
Trihydroxy-dimethoxy chalcone								Brazil [122]	
Tryhidroxymethoxy flavonon								Brazil [71]	
Vestitol	X			X		X		Mexico, Brazil [63,80,83]	[95,155]
Vestitone								Brazil [80]	
Violanthin						X		Brazil [122]	[173]
Xanthochymol								Brazil [83]	
ε-caprolactone derivative								Mexico [70]	

Only articles that reported the chemical composition of Brazil green and red propolis and North Mexican brown propolis were included in this table; moreover, we only included CAPE as a compound of Danish propolis due to the unique compound identified in the chemical composition of the work that evaluated the antiulcerative activity of this propolis [58]. Ga: gastroprotective; Au: antiulcerative; As: antisecretory; Af: anti-inflammatory; Im: immunomodulator; Ax: antioxidant; and Cp: cytoprotective.

## Data Availability

Not applicable.
